# Strain Differences in Bloodstream and Skin Infection: Methicillin-Resistant *Staphylococcus aureus* Isolated in 2018–2021 in a Single Health System

**DOI:** 10.1093/ofid/ofae261

**Published:** 2024-05-06

**Authors:** Katrina S Hofstetter, Natasia F Jacko, Margot J Shumaker, Brooke M Talbot, Robert A Petit, Timothy D Read, Michael Z David

**Affiliations:** Division of Infectious Diseases, Department of Medicine, Emory University, Atlanta, Georgia, USA; Division of Infectious Diseases, Department of Medicine, University of Pennsylvania, Philadelphia, Pennsylvania, USA; Division of Infectious Diseases, Department of Medicine, University of Pennsylvania, Philadelphia, Pennsylvania, USA; Division of Infectious Diseases, Department of Medicine, Emory University, Atlanta, Georgia, USA; Division of Infectious Diseases, Department of Medicine, Emory University, Atlanta, Georgia, USA; Division of Infectious Diseases, Department of Medicine, Emory University, Atlanta, Georgia, USA; Division of Infectious Diseases, Department of Medicine, University of Pennsylvania, Philadelphia, Pennsylvania, USA

**Keywords:** CC5, CC8, clonal complex, *Staphylococcus aureus*, whole-genome sequencing

## Abstract

*Staphylococcus aureus* is a common cause of skin and soft-tissue infections (SSTIs) and has become the most common cause of bloodstream infections (BSIs) in recent years, but whether the strains causing these two clinical syndromes overlap has not been studied adequately. USA300/500 (clonal complex [CC] 8–sequence type [ST] 8) and USA100 (CC5-ST5) have dominated among methicillin-resistant *S aureus* (MRSA) strains in the United States since the early 2000s. We compared the genomes of unselected MRSA isolates from 131 SSTIs with those from 145 BSIs at a single US center in overlapping periods in 2018–2021. CC8 MRSA was more common among SSTIs, and CC5 was more common among BSIs, consistent with prior literature. Based on clustering genomes with a threshold of 15 single-nucleotide polymorphisms, we identified clusters limited to patients with SSTI and separate clusters exclusively comprising patients with BSIs. However, we also identified eight clusters that included at least one SSTI and one BSI isolate. This suggests that virulent MRSA strains are transmitted from person to person locally in the healthcare setting or the community and that single lineages are often capable of causing both SSTIs and BSIs.

*Staphylococcus aureus* asymptomatically colonizes the nares of 28%–40% of the human population [1, 2]. *S aureus* is a common cause of skin and soft-tissue infections (SSTIs) [3] and in recent years has become the most common cause of bloodstream infections (BSIs) [4]. Nasal colonization of patients has been associated with an increased risk of both BSIs [5] and SSTIs [2, 3]. Colonization provides a reservoir from which *S aureus* can access the bloodstream after a minor skin injury, trauma, surgery, or a viral infection [3, 4].

Community-associated (CA) methicillin-resistant *S aureus* (MRSA) infections in the United States, epidemiologically defined as those occurring in individuals with no recent healthcare exposures, have been most often reported in younger, healthy individuals [3, 6]. Healthcare-associated (HA) MRSA infections, in contrast, are more likely to be diagnosed in older patients with comorbid conditions and are often invasive infections, such as BSIs or pneumonia [6]. Since at least 2004, the most often isolated MRSA strains in the United States have belonged to clonal complex (CC) 8 and CC5 [7].

USA300, which belongs to multilocus (ML) sequence type (ST) 8 (included in CC8), was initially associated with CA-MRSA infections, but since 2005 it has increasingly been recognized also as the cause of nosocomial infections [3, 6, 8]. USA300 has become the most common MRSA strain circulating in the United States [9]. USA500 is a closely related CC8 strain type that is easily distinguished from USA300 by whole-genome sequencing [10, 11]. USA100 (usually ST5, which belongs to CC5) is most often a HA-MRSA strain. However, it has also been uncommonly isolated from epidemiologically defined CA-MRSA infections and from nasal carriage in individuals with no prior healthcare exposures [12]. Thus, there are no strain types that consistently distinguish between epidemiologically defined CA-MRSA and HA-MRSA infections [13].

Reports from the last 15 years indicate that the majority of MRSA SSTIs in the United States have their onset in the community and are caused by USA300 [3, 7, 8]. One study of strains reported in the published literature found that in 2002–2013, 62% of SSTIs were caused by USA300 and 19% by the second most common strain type, USA100 [8]. The prevalence of USA300 among BSI isolates, however, may be increasing [14].

To understand the relationships between SSTI and BSI isolates, we performed whole-genome sequencing on SSTI and BSI MRSA strains from patients at two hospitals of the University of Pennsylvania from 2018 to 2021. We first compared the phylogenetic structure of MRSA isolates from SSTIs and BSIs. We then assessed whether any of these genomes were closely associated with one another, which would suggest recent transmission among study patients. Among the SSTI isolates alone, three clusters were identified. We also identified eight clusters containing strains from both SSTI and BSI cases, suggesting epidemiologic overlap and the spread of MRSA strains between patients with HA-MRSA and CA-MRSA infections within a single geographic area.

## METHODS

### Patient and Isolate Selection

MRSA isolates were collected by the Hospital of the University of Pennsylvania (HUP) Clinical Microbiology Laboratory from routine clinical specimens obtained from outpatient clinics, emergency departments, and inpatient units at HUP or the Penn Presbyterian Medical Center. All MRSA isolates from SSTIs were biobanked from December 2018 to February 2021. After screening of the electronic medical record (EMR) to document that a MRSA isolate was from an SSTI, each source patient was contacted and offered enrollment in the Study of the Evolution of MRSA, Antibiotics, Persistence Having the Outcome of Recurrence (SEMAPHORE), a National Institutes of Health–funded study to determine risk factors for recurrent *S aureus* infections during a 2-year follow-up period. Approximately 35% of eligible patients with SSTIs were enrolled prospectively in SEMAPHORE between December 2018 and February 2021 and were included in the present study. Separately, sequential patients with a MRSA BSI diagnosed from July 2018 to August 2020 were included in a retrospective study (the MRSA Bacteremia Retrospective Epidemiologic and Genomic Outcomes Study [BREGOS]) [15].

For both patients with SSTI and those with BSI, collection of demographic data from the EMR included age, race, sex, zip code of home residence, site of collection (emergency department, outpatient clinic, or inpatient), infection type, anatomic source of infection, epidemiologic classification (CA, HA, or HA community-onset [HACO] infection), and current intravenous drug use (IDU). Infections in patients whose first positive culture was obtained >48 hours after admission to a hospital were classified as HA-MRSA. If an infection occurred in an inpatient <48 hours after hospital admission or in an outpatient who had ≥1 specific healthcare exposure (hemodialysis, surgery, nursing home stay, or hospitalization within the past year or presence of an indwelling central venous catheter at the time of diagnosis), patients were classified as having an HACO-MRSA infection. Infections were classified as CA-MRSA if the patient had none of these healthcare exposures and was cultured as an outpatient or <48 hours after hospital admission. For patients with BSI, the Pitt Bacteremia Score and in-hospital mortality rate were recorded.

Participants in SEMAPHORE (those with SSTIs in the present report) each provided full, written informed consent for participation, and this was approved by the University of Pennsylvania Institutional Review Board (protocol 831208). Participants in the present study with bacteremia were enrolled in BREGOS with determination of an exemption for the need for individual informed consent after review by the University of Pennsylvania Institutional Review Board.

### DNA Sequencing

Isolates from the HUP Clinical Microbiology Laboratory were stored prospectively at −80°C. To prepare DNA for sequencing, frozen cultures were streaked on blood agar plates and incubated overnight at 37°C. Single isolates were passaged onto fresh blood agar plates before single colonies were isolated for sequencing. Isolates were sequenced at the Penn/Children's Hospital of Philadelphia (CHOP) Microbiome Center. Sequence libraries were prepared using the Illumina Nextera kit and sequenced by Illumina Hi-seq. Sequences for the SSTIs and BSIs are available in the National Center for Biotechnology Information Sequence Read Archive (PRJNA918392 and PRJNA751847, respectively).

### Bioinformatic Analysis

The Bactopia pipeline was used to assemble fastq files, call MLST and staphylococcal cassette chromosome *mec* (SCC*mec*) type, along with identifying variants [16]. CCs were assigned based on ST and PubMLST. If a CC was not available for an ST, the ST was used in place of the CC. All CC8 strains were classified as USA300 or USA500 based on the primer sequencing and the presence of genes coding for the Panton-Valentine leukocidin (PVL) genes and arginine catabolic mobile element (ACME) [10]. To generate maximum likelihood trees, core alignments were made using Parsnp software (version 1.7.4) [17], and trees were created using IQ-Tree software (version 2.2.0.3) [18]. The GGTREE package was used for visualization and figure generation [19]. Trees were rooted to GCF_000144955.1_ASM14495v1, and both USA300 (GCF_022226995.1_ASM2222699v1) and USA500 (GCF_016916765.1_ASM1691676v1) references were included. To identify clusters, we identified core genomes with <15 single-nucleotide polymorphism (SNP) differences, and pairwise distances were calculated using Disty McMatrixface software (https://github.com/c2-d2/disty). All R code and files are provided https://github.com/hofskatr/SSTI-BSI. We used the SCOARY2 tool (https://pubmed.ncbi.nlm.nih.gov/27887642/) with default parameters to assess for an association of any *S aureus* accessory gene with BSI or SSTI.

### Statistical Analysis

Patient and isolate characteristics were compared for SSTI and BSI cohorts. The χ^2^ test was used when testing for associations of demographic and clinical data with strain types with categorical data, unless sample sizes were small (<5 in a cell), in which case the Fisher exact test was performed. Comparisons of continuous data were made using the Wilcoxon rank sum test. All statistical analysis was performed using R software, version 4.2.2 [20].

## RESULTS

**Figure 1.**
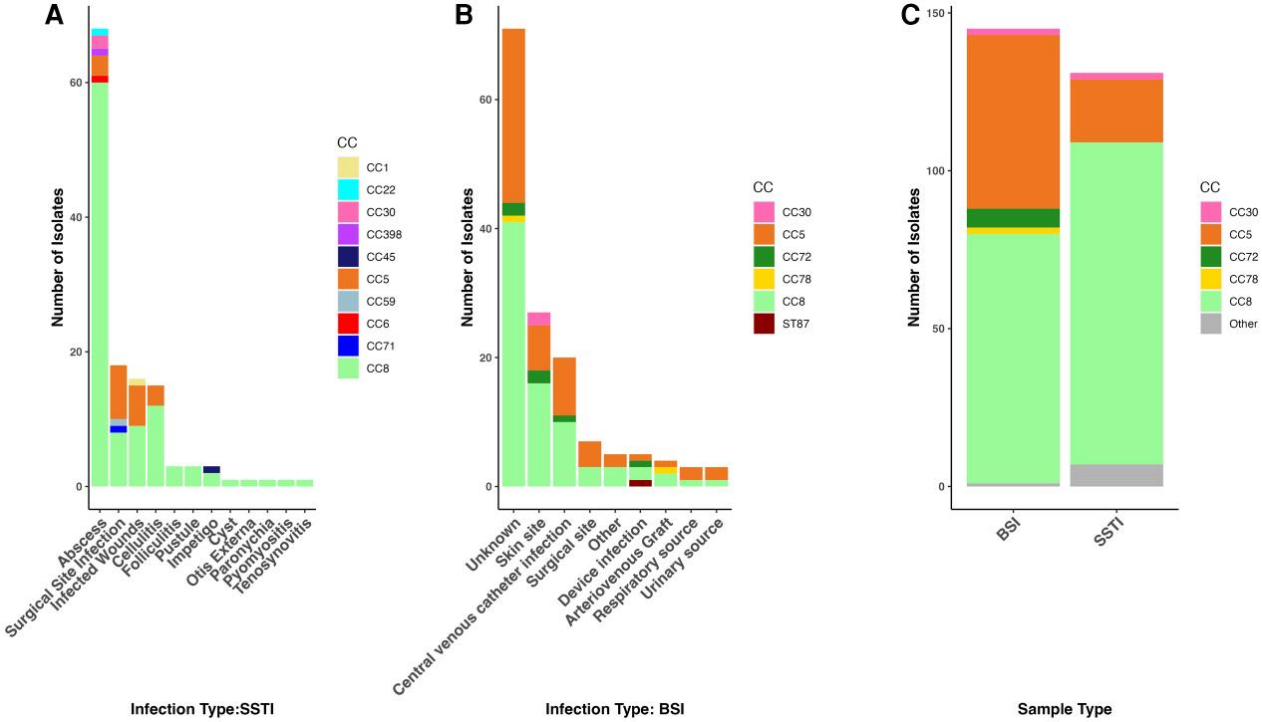
*A*, Distribution of infection types among skin and soft-tissue infections (SSTIs), stratified by clonal complex (CC). *B*, Distribution of infection types among bloodstream infections (BSIs), stratified by CC. *C*, CC distribution comparing SSTI and BSI strains. Abbreviations: AV, arteriovenous; CVC, central venous catheter.

During the study period, 131 patients with SSTIs were enrolled, and 145 sequential patients with a BSI were included in the BREGOS study [15]. Patients with SSTI and those with BSI did not differ significantly in distribution by age or race (Table 1 and Supplementary Figure 1). However, patients with SSTI were more likely to have CA-MRSA infections and less likely to have HA-MRSA or HACO-MRSA infections than those with BSI. Patients with SSTI were more likely to have had their infection diagnosed as an outpatient (46% vs 2%) or in the emergency department after which they were sent home (27% vs 3%) than those with BSI. BSIs were nearly all diagnosed in the inpatient setting (95%) (Table 1). Reported intravenous drug use was more common among patients with BSI (30 of 145 [21%]) than among those with SSTI (6 of 131 [5%]). Abscesses were the most common type of SSTI (52% [n = 68]), followed by surgical site infections (14% [n = 18]) and infected wounds (12% [n = 16]). In BSI cases with a known source of infection, the most common sources were a skin site (19%) or a central venous catheter infection (14%) (Table 2 and Figure 1). The in-hospital mortality rate for patients with BSI was 15% (22 of 145).

**Table 1.** Demographic and Clinical Characteristics of Patients with Methicillin-Resistant *Staphylococcus aureus* (MRSA) Infections and Clonal Complexes (CC) of the Causative MRSA Isolates, Comparing Bloodstream Infection and Skin and Soft-Tissue Infection Groups

Characteristic	Patients, No. (%)		*P* Value
	SSTI (n = 131)	BSI (n = 145)	
**Race**			
White	81 (62)	78 (54)	>.05
Black	43 (33)	58 (40)	
Asian	2 (2)	2 (1)	
Other	5 (4)	7 (5)	
**Sex**			
Female	62 (47)	70 (48)	>.05
Male	69 (53)	75 (52)	
**Epidemiologic type**			
HA	5 (5)	34 (23)	<.001
CA	64 (49)	17 (12)	
HACO	62 (47)	94 (65)	
**Site of diagnosis**			
ED	35 (27)	4 (3)	<.001
Inpatient	36 (27)	138 (95)	
Outpatient	60 (46)	3 (2)	
**Patient age**			
<50 y	52 (40)	57 (40)	>.05
>50 y	79 (60)	86 (60)	
**Current IDU**			
Yes	6 (5)	30 (21)	<.001
No	125 (95)	115 (79)	
**CC or ST**			
CC8	102 (78)	79 (55)	<.001
CC5	20 (15)	55 (39)	
CC1	1 (<1)	0	
CC6	1 (<1)	0	
CC22	2 (1)	0	
CC30	1 (<1)	2 (1)	
CC45	1 (<1)	0	
CC59	1 (<1)	0	
CC71	1 (<1)	0	
CC72	0	6 (4)	
CC78	0	2 (1)	
ST87	0	1 (<1)	

Abbreviations: BSI, bloodstream infection; CA, community associated; CC, clonal complex; ED, emergency department; HA, healthcare associated; HACO, HA community onset; IDU, intravenous drug use; SSTI, skin and soft-tissue infection; ST, sequence type.

**Table 2.** Types of Skin and Soft-Tissue Infections and Sources of Bloodstream Infection in the Current Study

Type or Source of Infection	Subjects, No. (%)
**Type of SSTI (n = 131)**	
Abscess	68 (52)
Surgical site infection	18 (14)
Infected wounds	16 (12)
Cellulitis	15 (11)
Folliculitis	3 (2)
Impetigo	3 (2)
Pustule	3 (2)
Otis externa	1 (<1)
Paronychia	1 (<1)
Cyst	1 (<1)
Pyomyositis	1 (<1)
Tenosynovitis	1 (<1)
**Source of BSI (n = 145)**	
Skin site	27 (19)
CVC infection	20 (14)
Surgical site	7 (5)
Device infection	5 (3)
Arteriovenous graft	4 (3)
Respiratory source	3 (2)
Urinary source	3 (2)
Other	5 (3)
Unknown	71 (49)

Abbreviations: BSI, bloodstream infection; CVC, central venous catheter; SSTI, skin and soft-tissue infection.

### Comparing Strain Types Among SSTI and BSI MRSA Isolates

SSTI isolates were assigned to 14 STs by genome-based MLST. CC8 and CC5 were the most abundant CCs among the SSTI isolates, accounting for the vast majority of all MRSA strains, with CC8 the most prevalent (102 of 131 isolates [78%]) and CC5 the second most prevalent (20 of 131 [15%]) (Table 1). Among abscesses, CC8 strains were by far the most common (60 of 68 [88%]; *P* > .01) (Figure 1). BSI isolates belonged to 5 CCs (CC8, CC5, CC72, CC78, and CC30), and 1 ST was not included in a CC (ST87). The BSI isolates were in 13 STs.

**Figure 2.**
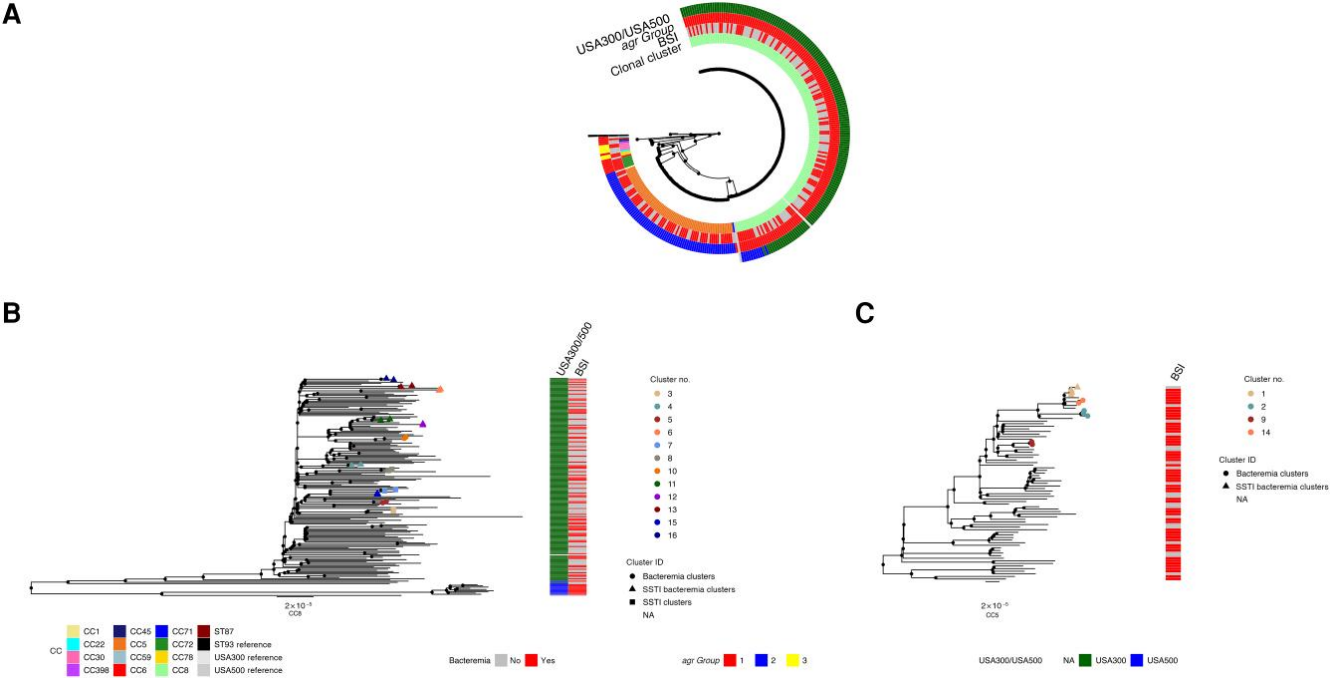
*A*, Maximum likelihood tree of all sequenced samples. Heat map depicts clonal complexes (CCs), whether the samples are from a bloodstream infection (BSI), and accessory gene regulator (*agr*) group. *B*, CC8 cluster only. *C,* CC5 cluster only; branch tips indicate clusters <15 single-nucleotide polymorphisms. Clusters with only BSI strains are represented with circles; clusters with only skin and soft-tissue infection (SSTI) strains, with squares; and clusters including both BSI and SSTI strains, with triangles. Abbreviations: ID, identification; NA, not applicable; ST, sequence type. Branch lengths are proportional to the number of nucleotide substitutions per site (scale bars of 2x10^−5^ are provided for reference).

We compared the genomes of the 131 SSTI isolates to 145 BSI isolates [15]. All of the CC8 strains were subtyped into either USA300 (101 SSTI and 69 BSI isolates) or USA500 [10] (1 SSTI and 10 BSI isolates). SSTI and BSI strains belonging to the same ST did not form separate subclades but instead were polyphyletic (Figure 2). For both SSTI and BSI isolates, the majority were CC5 and CC8 (Figure 1). The principal difference between the groups of strains was in the balance of the genotypes: CC8 strains were overrepresented in SSTIs (102 of 131 [78%]) compared with CC5 strains (21 of 131 [16%]) (Figure 1 and Table 1). CC5 strains were significantly more often found as a cause of BSIs (55 of 145 isolates [38%]) than of SSTIs (21 of 131 [16%]) (*P* < .001), while CC8 strains were more commonly a cause of SSTIs (102 of 131 [78%]) than of BSIs (79 of 145 [55%]) (*P* < .001) (Table 1 and Figure 1).

Comparing the antimicrobial susceptibilities of BSI and SSTI isolates, the patterns are quite similar. However, BSI isolates were significantly less likely to be susceptible to clindamycin (59% vs 73%; *P* < .05) and trimethoprim-sulfamethoxazole (93% vs 100%; *P* < .01) but did not differ significantly in resistance to erythromycin (11% vs 17%; *P* > .05) (Table 3). These associations were confounded by the different strain backgrounds associated with SSTI or BSI, and there was not a significant association between antimicrobial resistance genes and disease type (ie, BSI or SSTI) when adjusting for phylogeny, using the pangenome genome-wide association study tool SCOARY2 (Supplementary Table 2).

**Table 3.** Susceptibility of MRSA to Antimicrobial Agents, Comparing Skin and Soft Tissue Infection Isolates with Bacteremia (Bloodstream Infection) Isolates

Antimicrobial	SSTI Isolates (n = 131)		BSI Isolates (n = 145)		*P* Value
	No. Tested	Susceptible, No. (%)	No. Tested	Susceptible, No. (%)	
Ampicillin	130	0	145	0	NA
Cefazolin	130	0	145	0	NA
Penicillin	131	0	145	0	NA
Oxacillin	131	0	145	0	NA
Gentamicin	131	126 (96)	144	142 (99)	>.05
Ciprofloxacin	131	38 (29)	144	45 (31)	>.05
Levofloxacin	131	38 (29)	145	45 (31)	>.05
Moxifloxacin	131	39 (30)	144	46 (32)	>.05
Erythromycin	131	22 (17)	145	16 (11)	>.05
Clindamycin	131	95 (73)	145	86 (59)	<.05
Quinupristin-dalfopristin	130	130 (100)	144	144 (100)	NA
Linezolid	130	130 (100)	144	144 (100)	NA
Vancomycin	131	131 (100)	145	145 (100)	NA
Tetracycline	131	120 (92)	145	125 (86)	>.05
Tigecycline	131	128 (98)	145	139 (96)	>.05
Nitrofurantoin	131	129 (98)	144	144 (100)	>.05
Rifampicin	131	130 (99)	145	141 (97)	>.05
Trimethoprim-sulfamethoxazole	131	131 (100)	145	135 (93)	<.01
Daptomycin	0	0	130	130 (100)	NA
Ceftaroline	0	0	5	5 (100)	NA
Cefoxitin	130	0 (0)	144	2 (1)	>.05

Abbreviations: BSI, bloodstream infection; NA, not applicable; SSTI, skin and soft-tissue infection.

### Transmission Clusters Among SSTI and BSI Strains

While it has been reported that SSTIs can serve as a reservoir for BSIs, it is not known if MRSA causing BSIs and MRSA causing SSTIs, even within the same CCs, are distinct from one another. We thus investigated, among isolated genomes from our study, how closely strains from different patients with BSIs and SSTIs were related. Several studies have indicated that a 15-SNP difference threshold can be used to cluster isolates into epidemiologically linked groups that shared the same common ancestor in the past few years [15, 21–25]. In our combined group of 276 isolates from SSTIs and BSIs, we identified 16 clusters containing 2–4 isolates using the 15-SNP threshold, representing 13% of all isolates (35 of 276). All MRSA clones found in a cluster were either CC8 or CC5. The correlation of pairwise patristic distances between isolates on the phylogenetic tree and nucleotide sequence distances was 0.997 (Supplementary Figure 2).

Three clusters contained only strains from SSTIs (all CC8 strains), and in 5 clusters all strains were from BSIs (2 CC8 and 3 CC5 strains). Eight clusters contained at least 1 BSI and 1 SSTI isolate, including 7 CC8 and 1 CC5 cluster (Figure 2*B* and 2*C* and Supplementary Table 1). Comparisons of EMR data revealed that 1 cluster including both a BSI and an SSTI came from the same patient (cluster 15), while the remaining 7 came from different patients.

We compared a number of patient and isolate characteristics between clustered and nonclustered MRSA infections. We found that in 14 of 16 clusters there were no shared zip codes of residence for study patients. There were common zip codes only in clusters 4 and 15; however, cluster 15, as noted above, included only 2 isolates from the same individual. There were also no significant differences comparing patients with clustered or nonclustered MRSA clones among those with reported current intravenous drug use. Finally, there was no significant difference between either (1) CC8 and other CC types or (2) CC5 and other CC types when comparing clustered and nonclustered clones.

## DISCUSSION

The results of this study highlight divergent trends in infections caused by the two most common *S aureus* CCs currently isolated in the United States. CC8 isolates were strongly associated with SSTIs, and CC5 with BSIs. CC8 isolates that were subtyped to USA300 were the most common strain type in our study population.

The USA300 MRSA strain likely emerged in the early 1970s and spread rapidly during the 1990s and 2000s across North America to achieve its current predominant position among MRSA strain types [7, 26]. One study of 224 ST8 genomes suggested that the USA300 clone descended from an ST8 progenitor originating in central Europe in the 19th century, with an introduction to North America in the early 20th century, while gradually acquiring its typical genomic and virulence characteristics, such as PVL, SCC*mec* type IVa, and the ACME [27]. Another study of USA300 MRSA genomes suggested that the USA300 clone first emerged on the East Coast of the United States and perhaps in Pennsylvania [28].

Although USA300 MRSA was originally identified as a “CA-MRSA strain,” it was soon also recognized as a nosocomial pathogen, as early as 2005 [6, 8, 29, 30]. Indeed, USA300 has since become the leading cause of BSIs in the United States, which are usually classified as HA-MRSA or HACO-MRSA infections [4]. With this shift from the community to the healthcare setting, David et al [13] demonstrated among 616 sequential MRSA isolates from the University of Chicago Medical Center in 2004–2005 that there was already a poor correlation between the typical genotypic characteristics of CA-MRSA strain types—such as ST8, SCC*mec* type IV or PVL gene carriage, or lack of a multidrug resistance phenotype—and the Centers for Disease Control and Prevention's epidemiologic criteria for CA-MRSA infections.

In the current study, carried out in Philadelphia (750 miles from Chicago), and with isolates collected about 15 years later, the ST5 (USA100) and ST8 (USA300 or USA500) strains continue to dominate among MRSA isolates obtained from both blood and SSTIs. Many other US and Canadian studies with isolates collected between 2003 and 2018 have shown similar results, with a generally increasing percentage of BSIs and SSTIs caused by USA300/USA500, and a decreasing percentage caused by USA100 [14, 31–39]. In a cohort of 276 patients with MRSA SSTI or BSI at a single US institution in 2018–2021, CC5 and CC8 strains predominated. In fact, 93% of all SSTI strains were either CC5 or CC8, and CC8 strains were significantly more common among patients with SSTI than among those with BSI.

Many epidemiologic characteristics of patients and their MRSA isolates in the present study were consistent with the literature from the past 15 years, indicating only slowly shifting trends in relative strain prevalence during this period. Remarkably, we found that closely clonally related strains of MRSA caused both BSIs and SSTIs in different patients in our cohort, suggesting that the pathogenesis of invasive infections in very different human tissues may not require highly adapted strains. We do not yet know to what extent genetic differences between the CCs (eg, carriage of virulence genes such as ACME or PVL) play a role in the observed differences in sites of infection.

We found in this study that abscesses are the most common type of SSTI, and they were significantly enriched among the infections caused by CC8 strains, an observation consistent with previous literature on CA-MRSA strains [40]. Moreover, in patients >50 years old who presented with an SSTI, we observed a higher prevalence of CC5 strains than in younger patients. This trend could not be explained by differences in any specific infection type, although we did find that patients >50 years old in our cohort were more likely than younger patients to have surgical site infections and infected ulcers and wounds. The older population is likely to have had more HA risk factors than those <50 years old, and CC5 may be more prevalent in the older cohort because CC5 strains tend to cause infections more often in healthcare, rather than community, settings.

There have been few previous studies examining the genomes of MRSA infections from different anatomic sites at a single center. We identified potential MRSA transmission clusters using a conservative threshold of ≤15 SNPs. Three clusters were of only SSTI strains, suggesting local spread of MRSA clones among the patients. There were also 5 clusters including only BSI strains, representing probable healthcare transmission, as reported by Talbot et al [15]. Surprisingly, given that we showed that BSIs and SSTIs were commonly caused by different *S aureus* clones, we found that 50% of the clusters contained both disease types. This result suggested epidemiologic linkage between BSI and SSTI may be more frequent than previously realized. There is literature that discusses potential BSI reservoirs being concurrent SSTI [14] and colonization of the nares [5]. A prior SSTI within the last year also increased the risk of a BSI [14]. Thus, it is possible that some patients with BSI in our study became bacteremic after a MRSA SSTI; however, only a small number of patients had a known SSTI as a source of BSI in our cohort.

It is very likely that there was direct or indirect transmission of MRSA strains between study patients who were in a single cluster either in the community or in a healthcare facility at some time before their presentation with clinically apparent infections. While we did not find significant overlap in zip codes of residence within our genomic clusters, community transmission is not ruled out.

Limitations of our study include the relatively small size of the cohorts examined, the lack of epidemiologic data about potential interactions among individuals, and the fact that the study was performed only at a single center and thus may not be generalizable. Moreover, only 35% of patients with MRSA SSTI at our center were enrolled in the study, and it is possible that the cohort enrolled is not representative of all such patients. The enrolled patients with SSTI did not differ significantly in age distribution, sex, or race from patients with SSTI who were not enrolled. However, the enrolled group differed in the distribution of types of SSTIs; the enrolled group was more likely than the unenrolled group to have cellulitis, wound infections, or surgical site infections.

Our work highlights the importance of considering strain background in future studies of *S aureus* to understand the relative roles of strain and patient characteristics in determining sites of infection.

## Supplementary Data

Supplementary materials are available at *Open Forum Infectious Diseases* online. Consisting of data provided by the authors to benefit the reader, the posted materials are not copyedited and are the sole responsibility of the authors, so questions or comments should be addressed to the corresponding author.

## Supplementary Material

ofae261_Supplementary_Data
