# Supplementary figures and images for: Strain Differences in Bloodstream and Skin Infection: Methicillin-Resistant Staphylococcus aureus Isolated in 2018–2021 in a Single Health System
Source: Open Forum Infect Dis. 2024 May 6;11(6):ofae261. doi: 10.1093/ofid/ofae261 (PMC11160326; doi:10.1093/ofid/ofae261)

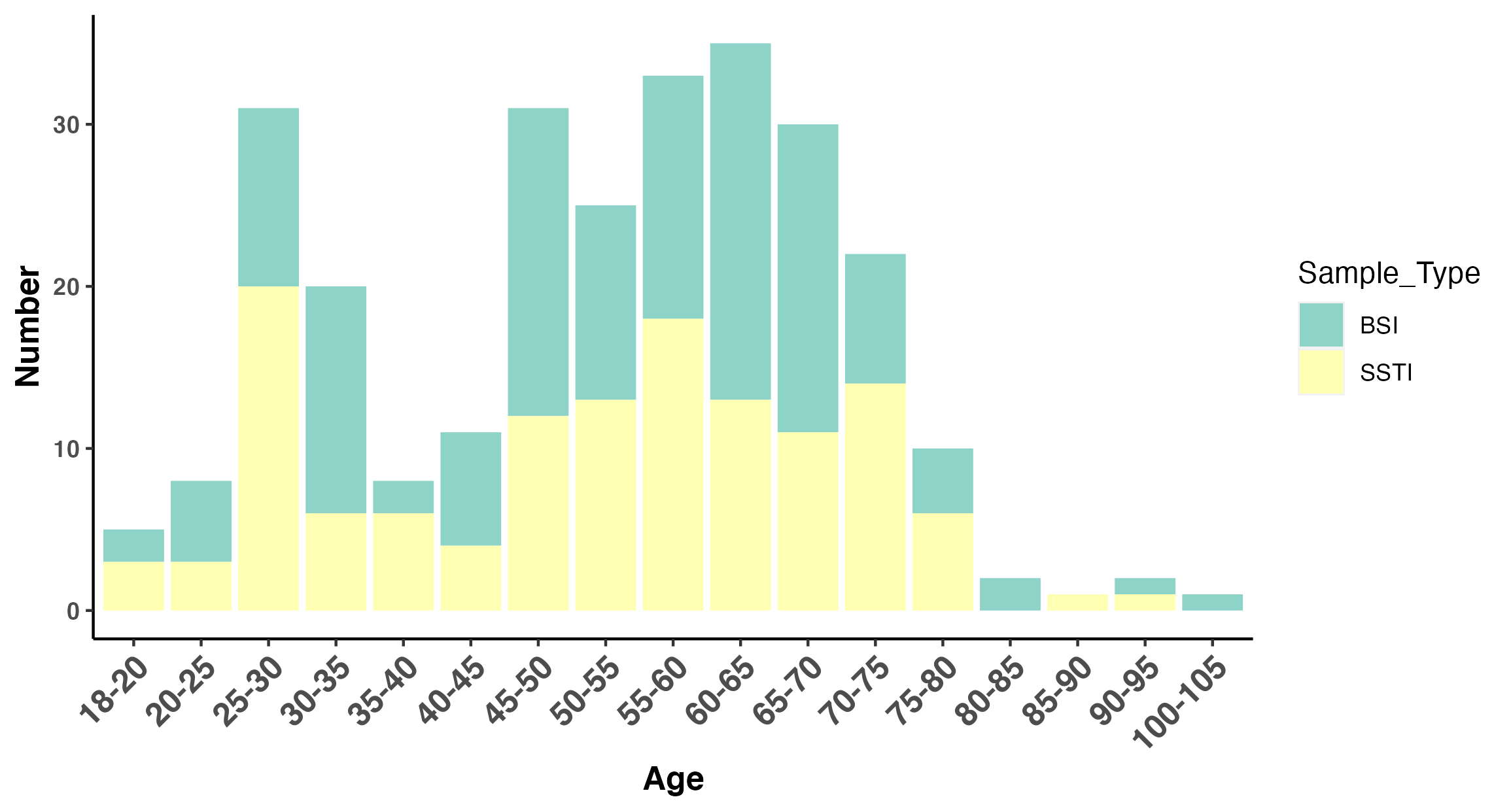

Supplement: ofae261_Supplementary_Data [file ofae261_supplementary_data.zip › supp_1.png]

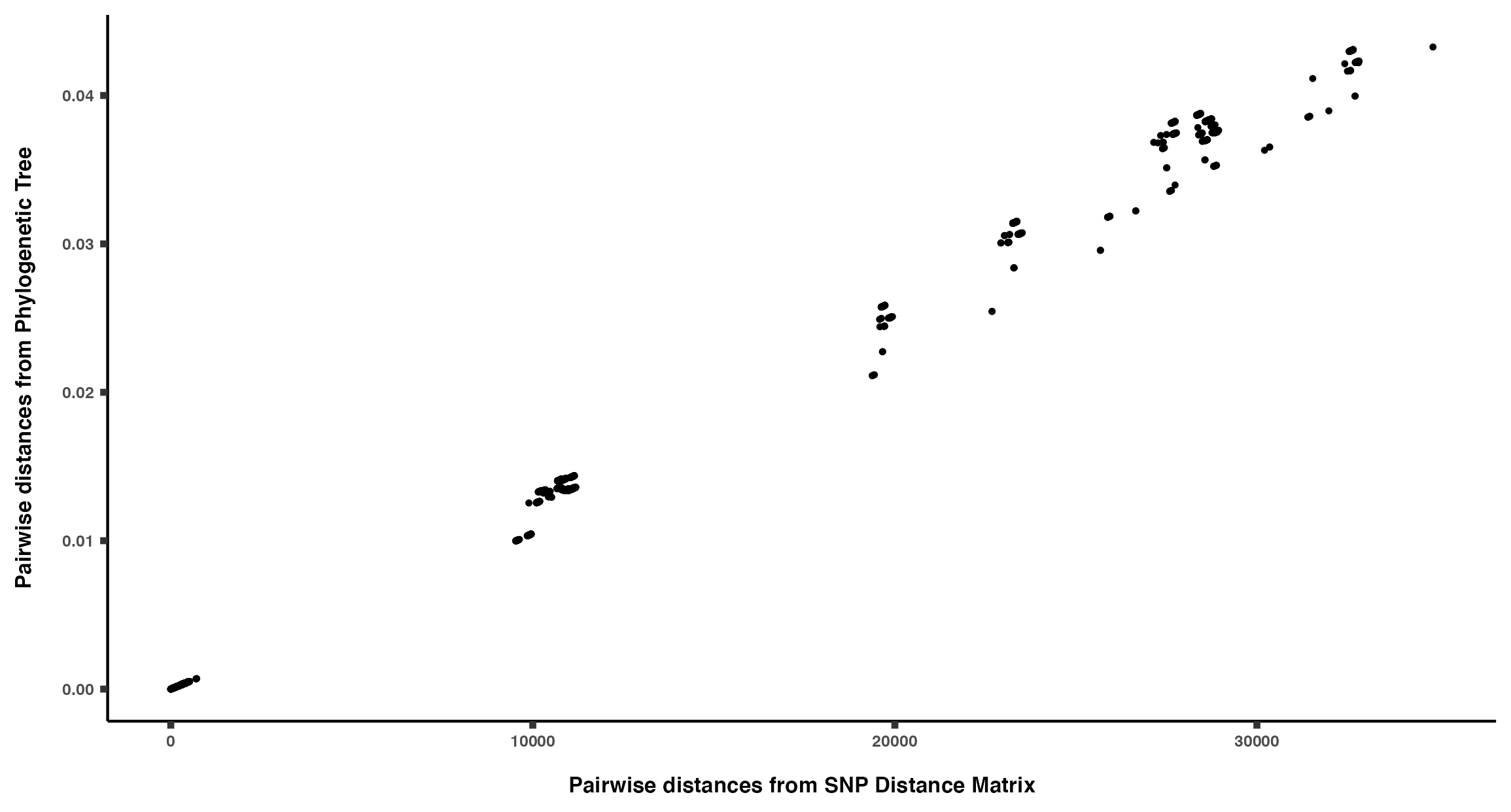

Supplement: ofae261_Supplementary_Data [file ofae261_supplementary_data.zip › supp_2.png]
